# A one-hour delayed school start improves sleep, sleepiness and inhibitory control in early adolescents in a randomized controlled trial

**DOI:** 10.1038/s41598-026-50892-6

**Published:** 2026-05-15

**Authors:** Eve Reynaud, Lucie Malevergne, Alexandre Grellet, Adrien Pawlik, Marc Gurgand, Amandine E. Rey, Stéphanie Mazza

**Affiliations:** 1 CNRS, INSERM, Centre de Recherche en Neurosciences de Lyon CRNL U1028 UMR 5292, FORGETTING Team, Université Claude Bernard Lyon 1, 69500 Bron, France; 2 Paris School of Economics, CNRS, and Ecole normale supérieure-PSL, Paris, France; 3 Department of Economics, SciencesPo, Paris, France; 4 J-PAL Europe, Paris School of Economics, Paris, France

**Keywords:** School start time, Sleep, Attention, Cognition, Adolescent, Actigraphy, Health care, Medical research, Neuroscience, Psychology, Psychology

## Abstract

Early school start times (SST) force adolescents to wake earlier than biologically preferred, creating chronic sleep debt. While evidence supports delaying SSTs, few controlled studies have used objective measures to assess their impact. We conducted a controlled intervention in a French boarding school to evaluate the effects of a one-hour delay in SST on sleep (N = 50) and cognitive functioning (N = 73) in early adolescents (age 12.8 years [11.7–14.2], 66% girls). After a baseline period with 8 a.m. starts (T0), four classes were randomized: half remained at the early schedule (Control-SST), half switched to 9 a.m. (Delayed-SST). Sleep measured by actigraphy, cognitive performance and mental health were assessed at baseline (T0) and 6 months later (T1). Between T0 and T1, total sleep time decreased in the Control-SST group, whereas the Delayed-SST group showed a modest, non-significant increase. This resulted at T1 in a 26-min between-group difference (Cohen’s d = 0.93, *p* = 0.007). Sleep onset time did not differ between groups. Sleepiness decreased in Delayed-SST but increased in Control-SST (d = -0.52, *p* = 0.042). Inhibitory control improved in Delayed-SST compared to Control-SST (d = -0.79, *p* = 0.001), with trends toward better sustained attention (d = -0.40, *p* = 0.051). Delaying SST by one hour appears to mitigate the progressive reduction in sleep duration commonly observed during adolescence, and benefits both cognitive functioning and sleepiness outcomes in early adolescence.

**Supplementary Information:**

The online version contains supplementary material available at 10.1038/s41598-026-50892-6.

## Introduction

Adolescence is a developmental period marked by significant biological and psychosocial changes, notably in the regulation of sleep and circadian rhythms. There is a natural tendency for sleep patterns to shift later during adolescence, a phenomenon known as a delay in chronotype or preferred sleep timing, primarily driven by pubertal maturation and its associated biological changes^1,2^. This shift results from both a slower accumulation of sleep pressure, part of the homeostatic regulation of sleep^3^, and a postponed onset of melatonin secretion, which reflects a shift in the internal circadian clock^4,5^. In parallel, adolescents experience growing autonomy from parental supervision and increasing exposure to evening social and academic activities, as well as screen time, all of which contribute to delayed sleep onset^6^. Early school start times (SST) lead to a mismatch between adolescents’ biological sleep preferences and imposed social schedules. The interaction of these biological and social influences results in a consistent reduction in total sleep time, a phenomenon that Carskadon and colleagues^7^ have described as “the perfect storm”. National surveys and longitudinal studies consistently confirm that sleep duration declines with age during adolescence^8–10^. Importantly, this reduction is not due to a decreased need for sleep but rather stems from the growing misalignment between delayed sleep onset and early school start^7^. As a result, adolescents frequently accumulate sleep debt during the week which they attempt to compensate on weekends, further disrupting their circadian rhythms^11^. This chronic sleep restriction is now recognized as a major public health concern. Insufficient sleep during adolescence has been linked to a host of negative outcomes, including increased daytime sleepiness, attention deficits, lower academic achievement, mood instability, behavioral issues, increased substance use, and even heightened risk for suicidal ideation and behaviors^12–14^.

In this context, delaying school start has emerged as a promising intervention to extend sleep duration and better align educational schedules with adolescents’ biological circadian rhythms^15–17^. In 2016, Wheaton and colleagues^15^ identified in their systematic review 38 articles investigating the effect of delaying school start on sleep, behavior, health and academic outcomes. They found consistent associations between later start times and longer sleep on weeknights, better school attendance, reduced tardiness, fewer instances of falling asleep in class, improved academic performance, and a decrease in motor vehicle accidents. However, most studies included were cross-sectional design, comparing schools with different start time, or before-after design without control groups, limiting causal inferences.

More recent quasi-experimental studies have assessed changes in outcomes before and after the implementation of delayed school start times and compared them to those of a control group with no schedule modification, thus assessing difference-in-differences^18,19^. This design strengthens causal inference by controlling for both pre-existing differences in sleep patterns and the natural developmental trajectories of adolescent sleep and cognition. For instance, Widome and colleagues^18^ took advantage of a district-led delay of 50 and 65 min in school start time at two high schools, while including three control schools from the same district that maintained their original 7:30 a.m. start time. Using actigraphy, they found that compared to the change observed in the control schools, students in the delayed-start schools gained on average 41 min of additional sleep on school nights after one year (CI_95%_ [25;57]) and 43 min after 2 years (CI_95%_ [25;61]). The authors reported minimal difference-in-differences regarding sleep onset time and sleep efficiency. This study provides strong evidence in favor of delaying school start, however the lack of randomization leaves open the possibility of residual confounding, such as baseline differences regarding sleep measures and demographic characteristics. However, conducting true randomized controlled trials remain logistically challenging^20^.

Thus, despite this growing body of research, gaps remain regarding controlled trials using objective measures of sleep and cognitive function. The present study sought to address this need by conducting a controlled trial in a school setting that was supportive of testing a delayed start time, evaluating the effect of a one-hour delay in school start time (from 8:00 a.m. to 9:00 a.m.) on: (1) sleep parameters, measured with actigraphy, (2) sleepiness assessed using a validated self-report scale and (3) cognitive functioning, including sustained attention and inhibitory control, measured using standardized tests. The study was carried out among early adolescents, in a boarding school setting, which provided a unique methodological advantage by minimizing inter-individual variability related to home environment, parental routines, and external social factors. Crucially, the absence of daily commuting eliminated a major source of variability in sleep and wake times often encountered in day school studies and eased logistical constraints.

## Methods

### Population and setting

The study was conducted in a public boarding school located in a rural area of France. All students resided at the school from Monday to Friday and usually shared a room with 3 or 4 other students. The school is part of a specialized program designed to support students from disadvantaged socio-economic backgrounds by providing a living environment conducive to studying, along with access to athletic and cultural extracurricular activities. The present study focused on early adolescence, in students in grades 7 and 8 (corresponding to the 2^nd^ and 3^rd^ year middle school in France), who are typically aged between 12 and 14 years old. Students in the last year of middle school were not included to avoid confounding effects related to the increased academic pressure of the national exam that is administered that year.

An a priori power analysis was conducted using R to estimate the sample size required to detect a minimum relevant between-group difference of 25 min in the change in total sleep time from baseline to follow-up^16^. Assuming a two-sided α of 0.05, 80% power, and a conservative estimate of variability derived from these previous studies, the required sample size was estimated at 17 participants per group (total N = 34).

**Fig. 1:**
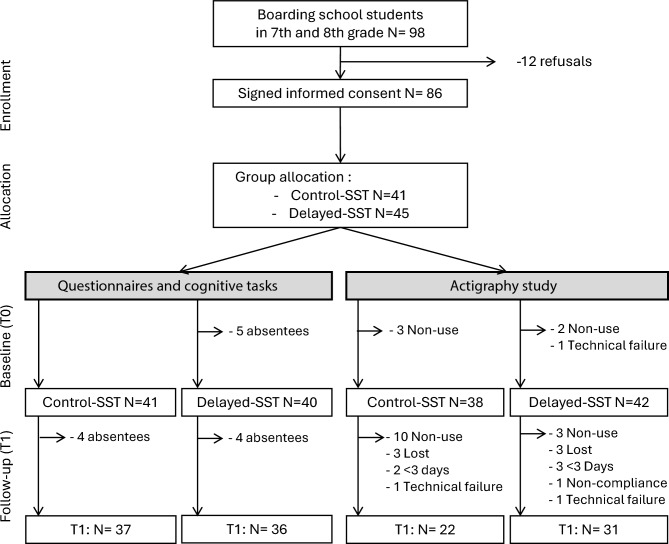
Flow chart. Participant flowchart illustrating enrollment, group allocation, and follow-up. A total of 98 students were initially considered, with 86 providing informed consent and allocated to either the control school start time (SST), with an 8 a.m. start throughout the year, or the Delayed-SST group, with a 9 a.m. school start following baseline measures. The figure details group sizes for the actigraphy study and the computerized tests separately.

The sample size is described in Fig. 1. Of the 98 students enrolled in the relevant grade levels, 86 agreed to participate in the study, and provided a signed informed consent from both the student and a parent or legal guardian. Data from 53 students were included in the actigraphy analyses, and 73 in the analyses based on computerized cognitive tests (see “Measures” Section). Inclusion in the actigraphy analyses required ≥ 3 days of valid recordings during school nights (Monday to Thursday) at both baseline (T0) and follow-up (T1). Actigraphy data were missing for 6 students at T0 (three from each group), due to device non-use (N = 5) or technical failure (N = 1). At T1, compliance substantially declined, resulting in the exclusion of 27 participants from the actigraphy dataset, including 16 from the control group. Reasons for exclusion at T1 were complete non-use (N = 13), insufficient wear time (N = 5), device loss (N = 6), and device malfunction (N = 2). One participant was excluded post hoc and treated as non-compliant in the analyses due to altered sleep patterns during Ramadan (pre-dawn waking for religious observance). Despite attrition in the actigraphy dataset, the final sample retained for sleep analyses remained above the minimum sample size estimated in the a priori power analysis (N = 34). Regarding the computerized cognitive tests, 5 students were absent during the T0 evaluation and 8 during the T1 evaluation. The flow chart of attrition is illustrated in Fig. 1.

### Study design

The study was conducted during the 2023–2024 school year and involved two 7th grade classes and two 8th grade classes. All methods were carried out in accordance with relevant guidelines and regulations. The study protocol was approved by the Paris School of Economics Institutional Review Board ethics committee (n° 2023–020). From September to the end of October, all students followed a standard schedule with classes starting at 8:00 a.m., Tuesday through Friday. Because all classes started at 10:00 a.m. on Mondays to accommodate students with long commutes, and this schedule was consistent across both groups and unrelated to the intervention, data from Sunday nights were excluded from the analyses. Of note, class ending remained the same, the delay in school start was compensated by limiting free periods. After a two-week vacation period, a delayed school start time intervention was implemented. Classes were randomly assigned by the school administrators to either the Delayed-SST group (for delayed school start time), in which school start times were delayed by one hour after the fall break (i.e. from early November), or the Control-SST group, which maintained an 8:00 a.m. start throughout the school year.

**Fig. 2:**
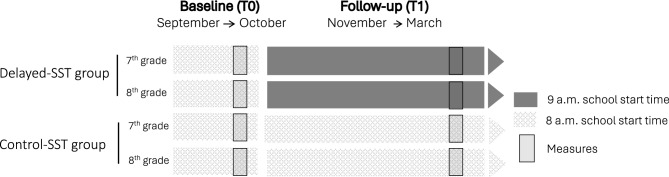
Study design and assessment timeline. Schematic of the study protocol at baseline (T0) and followup (T1) in control and delayed school start time groups including students from 7 and 8th grades. Students were assessed over 14-day actigraphy periods followed by questionnaires and cognitive testing. Light grey areas indicate periods during which school started at 8:00 a.m. (i.e., both groups at baseline and the control group at follow-up), whereas dark grey areas represent periods with a delayed school start time at 9:00 a.m. (intervention group at follow-up). Boxes indicate the timing of assessment sessions.

Baseline assessments (T0) were conducted between September and October, prior to the delayed school start intervention. During this time, students were instructed to wear an actigraph for two consecutive weeks. At the end of this phase, they completed a computerized evaluation including self-report questionnaires and cognitive tasks. These assessments were administered simultaneously by grade level using an online platform (Psytoolkit^21,22^) on the school’s computers. The same assessment procedure was repeated between February and March (T1). Computerized cognitive tests were conducted within fixed time windows (10:00–12:00 or 15:00–17:00) on the same day of the week at T0 and T1 to minimize time-of-day effects. These periods were selected to avoid circadian troughs and sleep inertia. The distribution of participants across testing sessions was balanced between groups. Chronotype was not used to schedule testing sessions. The study protocol is illustrated in Fig. 2.

The primary outcome of the study was total sleep time measured by actigraphy. Secondary outcomes included daytime sleepiness (assessed with the French Sleepiness Scale for Adolescents, FSSA), sustained attention (SART), and inhibitory control performance (Stroop task).

### Measures

#### Actigraphy

Sleep was assessed using actigraphy (Actiwatch 2; Philips-Respironics, Murrysville, USA, Philips Actiware software version 6.0.1), a wrist-worn device equipped with a piezoelectric accelerometer. The device samples activity data at a frequency of 32 Hz, aggregated into 60-s epochs. The threshold for sleep detection was set at the standard 40 activity counts per epoch^23^. Participants were instructed to press a button on the device to indicate the moment they went in bed, ready to sleep (Bedtime, BT, in decimal hours), and the moment they got out of bed (Rising Time, in decimal hours). When button presses were missing, BT and rising time were estimated using a combination of light and movement data from the device, supplemented by information from a sleep diary completed by the participants throughout the recording period. Sleep onset time (SOT, in decimal hours) was defined as the time at which sleep began, while Wake Time (WT, in decimal hours) was defined as the time sleep ended. Sleep Onset Latency (SOL, in minutes) was calculated as the interval between BT and SOT. Total sleep time (TST, in minutes) was defined as the duration between SOT and WT, minus any periods of Wake After Sleep Onset (WASO, in minutes). Sleep Efficiency (SE, expressed as a percentage) was calculated as TST divided by the time spent in bed (duration between BT and RT). Although students were asked to wear the actigraph continuously for 2 weeks, analyses focused exclusively on school nights (i.e. Monday through Thursday nights). While extended sleep duration on weekends may indicate adequate rest, it may also reflect compensatory sleep in response to accumulated weekday sleep debt^24^.

#### Questionnaires

Daytime sleepiness was evaluated using the French Sleepiness Scale for Adolescents (FSSA)^25^, an adaptation of the Epworth Sleepiness Scale for Children and Adolescents^26^. This self-report measure requires students to rate the likelihood of dozing off in eight different situations (e.g. while reading or sitting in a classroom). Each item is rated on a Likert scale ranging from 0 ("would never fall asleep") to 3 ("high chance of falling asleep"). The total score ranges from 0 to 24, with higher scores indicating greater levels of daytime sleepiness. A cutoff score strictly above 11 indicates acute sleepiness^25^.

Preference towards morningness or eveningness was assessed using the Morningness-Eveningness Scale for Children (MESC)^27^. It is a 10-item questionnaire, where participants indicate at what time they would rather engage towards various activities, such as waking up, taking an exam or doing sports. A higher score indicates a greater preference for morningness. Participants with a score strictly inferior to 23 were defined as having an evening chronotype, and others as a morning or neutral chronotype.

#### Cognitive functioning

Sustained attention was assessed using the “Sustained Attention to Response Task” (SART)^28^, a computerized go/no-go paradigm designed to measure impulsivity and ability to maintain attention over time. Participants are required to monitor visual displays acknowledging responses to frequent neutral signals (GO trials) but withholding response when detecting rare targets (NO-GO trials). Specifically, participants were instructed to press the space bar on the keyboard when a digit appeared on the screen (“go” trials) but to withhold their response when the presented digit was a “3” (“no-go” trials). Each digit was displayed for 250 ms (ms), followed by a visual mask lasting 900 ms. The task included 18 practice trials, followed by 225 experimental trials, comprising 200 “go” trials and 25 “no-go” trials for a duration of approximatively 7 min. Omission errors were defined as failures to respond during a go trial, while commission errors were defined as responses made during no-go trials. Reaction times (RTs) were computed only for correct go trials. To minimize the influence of extreme values, which are likely fast guesses without cognitive processing, a within-subject low-pass filter was applied to the RT data. Specifically, RTs faster than two standard deviations below the individual’s mean RT were excluded (0.28% of RT data was removed). The task duration was in average 5.6 (SD = 0.8) minutes.

Students also completed a computerized Stroop task^29^, which provides a measure of inhibitory control, selective attention and processing speed^30^. In this task, participants were instructed to identify the ink color in which a word was printed (“red,” “green,” “blue,” or “yellow”) and to ignore the semantic content of the word itself. Each response was required within 2000 ms of stimulus presentation. Following a 10-item training session, the experimental phase consisted of 100 trials: 25 congruent trials, in which the ink color matched the word meaning, and 75 incongruent trials, in which the word’s meaning corresponded to a different color. The Stroop effect is defined as the difference in mean reaction time (RT) between incongruent and congruent trials, with greater RT differences indicating lower inhibitory control. Only correct responses were included in RT analyses. Within-participant RT outliers, defined as RTs faster than two standard deviations below the participant’s mean, were excluded, accounting for approximately 1.2% of the trials. On average, this task lasted 3.7 (SD = 0.24) minutes.

#### Socio-demographic data

The school provided information about the participants’ age, gender (also confirmed within the computerized questionnaire) and scholarship status, which was used as a proxy for socio-economic status. Scholarships are based on the income of the person or persons responsible for the student and the number of dependent children (not on academic performance).

### Analyses

All analyses were non-parametric and conducted using R sofware^31^. First, to assess differential attrition, a comparison between included and excluded participants was conducted to understand selection bias regarding gender, age, socio-economic status and group allocation. Secondly, we assessed the comparability of the included participants between intervention groups (Control-SST vs Delayed-SST) at baseline, where we compared gender, age, socio-economic status, as well as all actigraphy, questionnaires and cognitive functioning. Then, the difference between baseline and follow-up (T1-T0) was calculated for all outcome measures and compared between groups and referred to as difference-in-differences analyses. This methodology allows to control for baseline differences between groups and for the natural change that occurs when no intervention is conducted. We hypothesized that the delay in SST would benefit more participants with an evening chronotype and thus conducted a complementary analysis stratifying by chronotype (MESC < 23). We also examined the correlations between changes in total sleep time between baseline and follow-up (T1-T0) and changes in daytime functioning (FSSA, SART and Stroop). For all comparisons, Mann-Whitney U-tests were used to compare the distribution of continuous variables, chi-square to compare binary outcomes, and Spearman’s or Pearson’s correlation coefficients to examine associations. A two-tailed *p* < 0.05 was considered statistically significant. As a measure of precaution since students are grouped in class, we calculated the Intraclass Correlation Coefficients (ICC), to estimate if considering clustering in the analyses was necessary. An ICC close to zero implies no clustering of the data. The ICC of sleep duration at T0 was equal to -0.033 CI_95%_[ -0.042 ; + 0.904]. Due to the small sample size and number of clusters, the calculation of the ICC presents approximation, with a wide confidence interval and a negative ICC. Negative ICCs are conventionally treated as zero^33^, therefore, it was deemed unnecessary to account for within-group clustering in the analyses.

## Results

### Population

Questionnaires and cognitive measures were available for the pre and post intervention conditions in 73 students (85% of the participants). After applying criteria for actigraphy data validity (at least three days of usable recording), we retained usable sleep data from 50 participants with pre and post intervention measures. Importantly, participants with and without usable data did not differ significantly in terms of age, gender, socio-economic status, or experimental group (see supplementary table).

### Actigraphy study

#### Baseline

At baseline (T0), groups were not significantly different regarding socio-demographic characteristics (Table 1), with an average age of 12.8 years (SD = 0.67), 68% were girls and 48% benefited from a scholarship. Similarly, no differences were observed in objectively measured sleep on school nights, with an average sleep onset occurring at 9:50 p.m. (SD = 12 min), a rising time at 6:05 a.m. (SD = 15 min) and a total sleep time of 7 h and 20 min (SD = 37 min).

**Table 1:** Baseline comparison between groups regarding demographic data and school-night sleep (measured by actigraphy).

	Control-SST group (N = 20)	Delayed-SST group (N = 30)	*p*^a^
Age	12.9 (0.7)	12.7 (0.7)	0.312
Girl (yes)	70% (14)	66.7% (20)	0.804
Scholarship (yes)	55% (11)	43.3% (13)	0.419
Bedtime (hh:mm)	21:33 (0:17)	21:34 (0:08)	0.272
Sleep onset time (hh:mm)	21:50 (0:17)	21:50 (0:12)	0.411
Wake time (hh:mm)	6:02 (0:21)	6:08 (0:10)	0.378
Rising time (hh:mm)	6:16 (0:22)	6:20 (0:08)	0.797
Total sleep time (hh:mm)	7:20 (0:18)	7:23 (0:25)	0.589
Sleep onset latency (min)	16.8 (7.3)	16.4 (8.1)	0.566
Wake after sleep onset(min)	51.6 (15.0)	55.2 (16.4)	0.851
Sleep efficiency (%)	84.2 (3.7)	84.2 (4.2)	0.589

^a^ Mann Whitney U test and Chi-square for the 2 binary variables: gender and scholarship status. SST: School Start Time.

#### Difference-in-differences

**Fig. 3:**
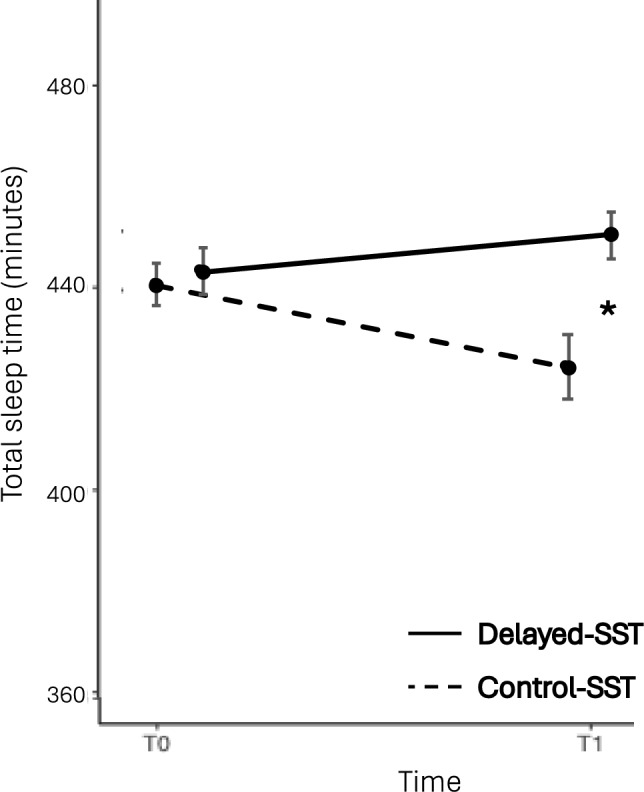
Differences-in-differences in total sleep time by time and group. Mean total sleep time (TST) at baseline (T0) and follow-up (T1) in the control-SST (dashed line) and delayed-SST (solid line) groups. Error bars represent standard errors of the mean. *p* < 0.05.

Difference-in-differences analysis for sleep outcomes are presented in Table 2. Between T0 and T1, total sleep time (TST) significantly decreased in the Control-SST group by 15.6 min (SD = 28.9, *p* = 0.046), whereas a modest but non-significant increase was observed in the Delayed-SST group (+ 7.2 min, SD = 21.1, *p* = 0.092). This resulted in a significant between group difference-in-differences estimate of + 22.8 min (CI_95%_[+ 7.6 ; + 37.9], Cohen’s d = 0.93, *p* = 0.007). At T1, adolescents in the Control-SST group had on average 7 h and 4 min of sleep per night (SD = 28 min), while those in the Delayed-SST group slept 7 h and 30 min (SD = 24 min), corresponding to a significant 26-min between-group difference in absolute sleep duration at T1 (CI_95%_[+ 10 ; + 43], illustrated in Fig. 3). In the Control-SST group, 50% of the students slept less than 7 h per night, compared to 13% in the Delayed-SST Group (X^2^ = 6.3, *p* = 0.012).

**Table 2:** Difference-in-differences changes in sleep between baseline and follow-up (T1-T0) according to the intervention group.

	T1-T0 differences (mean ± SD)^a^				Difference-in-differences^c^	Cohen’s d	*p*-value^d^
	Control-SST group	*p*^b^	Delayed-SST group	*p*^b^	[95% CI]		
Bedtime (min)	+ 13.6 ± 27.9	0.021	+ 4.7 ± 26.4	0.105	-8.9 [-24.7 ; + 7.0]	-0.33	0.520
Sleep onset time (min)	+ 14.0 ± 27.6	0.015	+ 8.0 ± 28.9	0.036	-6.0 [-22.3 ; + 10.3]	-0.21	0.806
Wake time (min)	+ 10.7 ± 32.4	0.145	+ 23.3 ± 25.9	< 0.001	+ 12.6 [-4.8 ; + 30.0]	0.44	0.031
Rising time (min)	+ 11.5 ± 28.4	0.040	+ 27.7 ± 21.8	< 0.001	+ 16.2 [+ 1.1 ; + 31.2]	0.66	0.001
Total sleep time (min)	-15.6 ± 28.9	0.046	+ 7.2 ± 21.1	0.092	+ 22.8 [+ 7.6 ; + 37.9]	0.93	0.007
Sleep onset latency (min)	+ 0.4 ± 7.3	0.575	+ 3.3 ± 9.7	0.109	+ 2.9 [-1.9 ; + 7.7]	0.33	0.382
Wake after sleep onset (min)	+ 12.3 ± 21.4	0.033	+ 8.1 ± 16.4	0.011	-4.2 [-15.6 ; + 7.2]	-0.23	0.822
Sleep efficiency (%)	-2.6 ± 4.9	0.036	-2.1 ± 3.6	0.003	+ 0.5 [-2.1 ; + 3.0]	0.11	0.914

^a^ “ + ” indicates an increase between T1 and T0, ^b^ Paired samples Wilcoxon ranked test, ^c^ “ + ” indicates a greater increase (or a lesser decrease) between T1 and T0 for the Delayed-SST group than for the control group, ^d^ Mann–Whitney U test of the difference-in-differences.

**Fig. 4:**
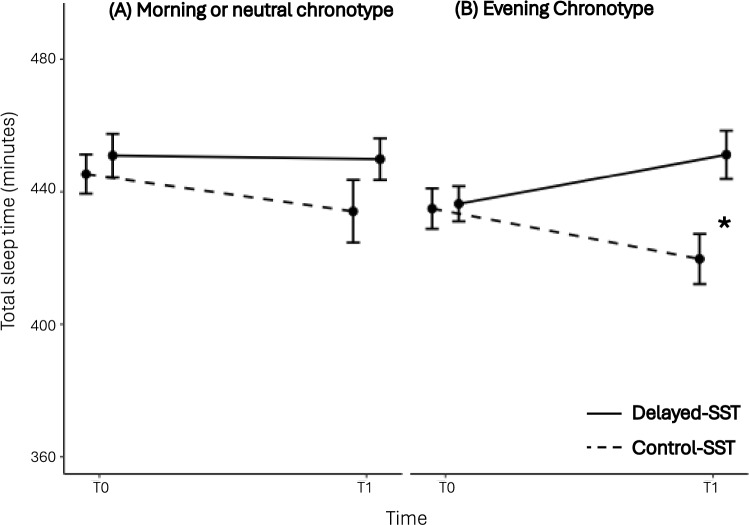
Differences-in-differences in total sleep time by time, group and chronotype. Panel (**A**) Mean total sleep time (TST) at baseline (T0) and follow-up (T1) in the control-SST (dashed line) and delayed-SST (solid line) groups within adolescents with morning or neutral chronotype (MESC ≥ 23). Panel (**B**) Mean total sleep time (TST) at baseline (T0) and follow-up (T1) in the control-SST (dashed line) and delayed-SST (solid line) groups within adolescents with an evening chronotype (MESC < 23). Error bars represent standard errors of the mean. *p* < 0.05.

The evolution of sleep onset time between T0 and T1 did not significantly differ between the two groups (difference-in-differences -6.0 min CI_95%_[− 22.3 ; + 10.3], *p* = 0.806). In contrast, wake time was significantly delayed by 12.6 min in the Delayed-SST group compared to the Control-SST group (CI_95%_[− 4.8 ; + 30.0], *p* = 0.031). Interestingly, when participants were stratified by chronotype, adolescents with an evening chronotype showed a significant TST difference-in-differences of 28.5 min between the Delayed-SST and Control-SST groups (95% CI [+ 8.8 ; + 52.7], *p* = 0.004), while no significant difference was found for adolescents with a morning or neutral chronotype (9.6 min, 95% CI [− 15.0 ; + 35.2]; see Fig. 4). These findings suggest that delaying school start time may be particularly beneficial for adolescents with biologically later sleep preferences.

### Questionnaires and cognitive tests

#### Baseline

At baseline, no significant differences were observed between the two groups in self-reported sleepiness (FSSA) see Table 3. Overall, 25% of participants scored above the clinical cutoff for excessive sleepiness (FSSA). In the SART, students in the Control-SST group made significantly more commission errors than those in the Delayed-SST group at baseline (respectively 15.7, SD = 5.9 vs 12.7, SD = 5.6, *p* = 0.026). They also exhibited a tendency towards faster reaction time (181.62 ms, SD = 83.77 vs 221.44 ms, SD = 94.86) compared to the Delayed-SST group (*p* = 0.064). Regarding cognitive performances, no significant group differences were found on the STROOP test, assessing inhibitory control, selective attention and processing speed.

**Table 3:** Baseline comparison between groups regarding questionnaires and cognitive functioning.

	Control-SST Group (N = 37)	Delayed-SST Group (N = 36)	p^a^
FSSA	9.73 (3.37)	8.78 (3.73)	0.451
SART			
Commission errors	15.70 (5.87)	12.70 (5.55)	0.026
Omission errors	11.81 (11.85)	11.18 (9.47)	0.850
Reaction time (ms)	181.62 (83.77)	221.44 (94.86)	0.064
STROOP effect (ms)	81.10 (69.39)	115.23 (87.99)	0.086

Standard errors are indicated in parentheses.

^a^Mann–Whitney U test.

#### Difference-in-differences

Difference-in-differences analyses for the questionnaires and cognitive tests are detailed in Table 4. Between T0 and T1, students in the Control-SST group showed a significant increase in sleepiness score of 2 points on the FSSA scale (SD = 3.5) whereas no statistically significant change was observed in the Delayed-SST group (0.0, SD = 4.1), resulting in a medium effect size (Cohen’s d = 0.52, *p* = 0.042).

**Table 4:** Difference-in-differences changes in questionnaires and cognitive functioning between baseline and follow-up (T1-T0) according to the intervention group.

	T1-T0 differences (mean ± SD)^a^				Difference-in-differences^c^ [95% CI]	Cohen’s d	*p*-value^d^
	Control-SST group	*p*^b^	Delayed-SST group	*p*^b^			
FSSA	2.0 ± 3.5	0.001	0.0 ± 4.1	0.610	-2.0 [-3.7 ; -0.2]	-0.52	0.042
SART							
Commission errors	-2.2 ± 6.7	0.081	-4.5 ± 4.8	< 0.001	-2.3 [-5.0 ; + 0.4]	-0.40	0.051
Omission errors	-4.1 ± 12.4	0.068	-2.1 ± 7.3	0.062	1.9 [-2.8 ; + 6.7]	0.19	0.899
Reaction time (ms)	+ 2.4 ± 79.3	0.470	-2.9 ± 71.0	0.160	-5.4 [-40.5 ; + 29.7]	-0.07	0.554
STROOP effect	+ 17.9 ± 71.1	0.158	-49.9 ± 99.5	0.005	-67.9 [-108.3 ; -27.4]	-0.79	0.001

^a^ “ + ” indicates an increase between T1 and T0.

^b^Paired samples Wilcoxon ranked test.

^c^ “ + ” indicates a greater increase (or a lesser decrease) between T1 and T0 for the Delayed-SST group than for the control group.

^d^ Mann–Whitney U test of the difference-in-differences.

Performances on the Sustained Attention Response Task (SART) indicated a significant reduction in commission errors in the Delayed-SST group (− 4.5 errors, SD = 4.8, *p* < 0.001) but not in the control group (− 2.2, SD = 6.7, *p* = 0.081). This group-by-time difference approached statistical significance (difference-in-differences =  − 2.3; 95% CI [− 5.0; + 0.4], *p* = 0.051) suggesting a trend toward improved impulsivity control following the intervention. No significant group differences were obtained in changes in omission errors or in reaction times (respectively d = 0.19, *p* = 0.899 and d =  − 0.07, *p* = 0.554).

Regarding the Stroop task, performance did not significantly change in the Control-SST group between T0 and T1 (+ 17.9 ms, SD = 71.1, *p* = 0.158), whereas performance improved in the Delayed-SST group (− 49.9 ms, SD = 99.5, *p* = 0.005). The resulting difference-in-differences was 67.9 ms (95% CI [− 108.3; − 27.4], Cohen’s d =  − 0.79, *p* = 0.001), reflecting a significant improvement in inhibitory control and selective attention following the delayed school start.

Exploratory correlation analyses showed no significant association between individual changes in total sleep time from T0 to T1 and changes in daytime sleepiness (FSSA), sustained attention, or inhibitory control (all *p* > 0.05). This suggests that, in the present sample, the beneficial effects observed at the group level were not linearly related to inter-individual variations in sleep changes.

## Discussion

This controlled study evaluated the impact of a one-hour delay in school start time on sleep patterns, cognitive functioning and daytime sleepiness in early adolescents. Our findings demonstrate significant benefits of this intervention on multiple outcomes, reinforcing the growing body of literature advocating for later school start times for adolescents.

Objective measurements of sleep revealed that delaying school start times by one hour appears to counteract the progressive reduction in sleep duration commonly observed during adolescence^9^. While students in the Control-SST group exhibited a decline in sleep duration over the six-month period (-15.6 min), those in the Delayed-SST group showed a modest gain (+ 7.2 min), yielding a difference-in-differences estimate of 23 min. This translated into a cross-sectional difference of 26 min in total sleep time between groups at T1. This result closely aligns with the meta-analysis by Bowers et al.^17^ which reported an average gain of 20 min in total sleep time across longitudinal studies implementing delayed school start times (weighted effect size of d = 0.38, *p* < 0.001). Importantly, the magnitude of benefit appears to scale with the extent of the delay, with longer delays generally associated with greater increases in sleep duration. However, Bowers et al.^17^ were unable to evaluate potential moderators such as sex, age or the type of sleep measures (subjective vs objective) due to insufficient reporting or limited variability across studies. Given the impossibility of blinding school start time to participants, the reliance on self-reported sleep data may create expectancy effects and social desirability bias potentially inflating estimated effect sizes.

Our findings suggest that the benefits of later school start times are already observable in early adolescence, before the full onset of pubertal circadian shifts. This developmental period is characterized by neurobiological changes, including a progressive delay in melatonin secretion^5^ and a shift toward evening sleep–wake preferences^32^, which progressively increases the misalignment between adolescents’ biological rhythms and early school schedules. However, changes in sleep patterns during this stage are likely multifactorial and may also reflect contextual influences such as increasing academic demands across the school year. Our data further support this interpretation showing that early adolescents with an evening chronotype benefited most from the delayed start time. As circadian phase delay intensifies throughout puberty, the benefits of later start times are likely to become more pronounced with age^33^. Further studies are needed to better characterize how pubertal maturation, from early through late adolescence, modulates the effects of delayed start times on sleep and daytime functioning.

Importantly, we observed that delayed school start time did not significantly affect sleep onset time. This finding challenges the common concern that later school start times simply lead adolescents to go to bed later, thereby negating potential benefits. Instead, the preserved bedtime combined with later wake times resulted in extended sleep duration. This pattern has been consistently reported in prior studies^18,34–37^. In parallel with this objective increase in sleep duration, subjective sleepiness was also improved. While students in the control group reported increased sleepiness over time, those in the intervention group maintained stable levels, resulting in a medium effect size difference between groups. This result confirms the consistently reported reduction in daytime sleepiness observed in delayed school start studies^17^.

Exploratory correlation analyses in the present study did not show significant associations between individual changes in total sleep time and changes in sleepiness. This may suggest that the effects of delayed school start time on daytime functioning are not exclusively mediated by the magnitude of sleep changes but may also involve other factors such as reduced morning stress, better circadian alignment, or improved sleep regularity.

Few studies have directly assessed the effect of delayed school start times on standardized cognitive measures. Alfonsi and colleagues^38^ demonstrated that students starting at 8:00 am exhibited progressive declines in psychomotor vigilance performance across the academic year, while those starting at 9:00 a.m. maintained stable performance, supporting the role of delayed school start in preserving sustained attention. Lufi et al.^39^ also reported improvement in sustained and selective attention after 2 weeks of delayed school start time. In our study, the intervention was also associated with improvement in cognitive functioning. We observed a significant reduction in the Stroop interference effect, with a robust difference-in-differences effect size (d =  − 0.79), indicating improved inhibitory control and selective attention in the Delayed-SST group. Similarly, a trend toward improved performance was also observed in the SART, with reduced commission errors, suggesting improved impulsivity control. These findings are in line with previous research suggesting that prefrontal cognitive functions are particularly sensitive to variations in sleep duration during adolescence^40^. These experimental findings are further reinforced by converging real-world behavioral evidence showing that adolescents with insufficient or poor sleep-quality are more susceptible to impulsivity-related outcomes. These include higher sensation-seeking tendencies, increased transport risk taking, greater involvement in violent or delinquent behaviors and increased substance use^41^. Taken together, this body of evidence highlights the critical role of sleep in supporting cognitive control and psychosocial development during adolescence. Nevertheless, individual changes in total sleep time were not significantly correlated with changes in sustained attention or inhibitory control in the present sample. Given the modest sample size, this absence of association should be interpreted cautiously and does not preclude more complex or non-linear pathways linking sleep extension to cognitive functioning.

Although we did not assess the impact of the intervention on academic performances, some studies have observed positive associations between later school start times and improved academic performance^19,42,43^. However, the systematic review by Biller and colleagues^44^, concluded that no generalizable improvement in academic achievement can be drawn due to substantial heterogeneity in academic outcomes, study designs and delay durations. Focusing on peer-reviewed longitudinal studies with a control group, three reported no change in global academic outcomes^43,45,46^ (such as grade point average), one found a GPA improvement after 2 years^19^, and others reported subject-specific improvements (such as reading or math)^42,43^. Authors underscore the limitations of using academic test scores alone as indicators of the success or failure of such interventions^44,45^.

We did not include standardized measures of mental health symptoms in the present study, which limits the ability to evaluate the impact of delayed school start time on emotional functioning. However, longitudinal studies have linked insufficient sleep and poor sleep quality to increased anxiety and depressive symptoms in adolescents^47^. Previous studies have also reported improvement in mood^48^, well-being^49^ and depressive symptoms following a delayed school start time^35,36,50^. Similarly, sleep-based interventions have also shown improvement in anxiety among adolescents^51^. For example, Blake and colleagues^52^ conducted a randomized control trial implementing a cognitive-behavioral based sleep intervention in 123 adolescents, and found improvement in anxiety symptoms, although not in depressive symptoms.

Despite the demonstrated efficacy of the intervention, the average sleep duration in the delayed-SST group remained relatively short for adolescents. This aligns with previous findings that even when structural changes are implemented, a substantial proportion of adolescents continue to obtain insufficient sleep. Bowers et al. reported in their meta-analysis^17^ a post intervention average sleep duration of 7 h and 24 min. The present intervention partially reduced the prevalence of chronically sleep deprived students, with 50% in the Control-SST group sleeping less than 7 h compared to 13% in the Delayed-SST Group at follow-up. Sleep duration estimates in the present study were derived from actigraphy, which provides an objective and typically more conservative measure of sleep than self-reported assessments, notably because it accounts for nocturnal awakenings. This methodological consideration should be considered when interpreting sleep duration values and when comparing findings across studies or with international sleep duration recommendations, which are largely derived from self-reported data^53^.

However, even if this reduction in the prevalence of short sleep is meaningful, it indicates that later start times alone are insufficient. These results highlight the need to consider additional structural and cultural measures alongside start time adjustments to promote adequate sleep in adolescents. Complementary interventions, such as limiting evening electronic device use^54^, limiting late extracurricular activities and regulating electronic assignment submission times, may be necessary to foster a school environment that is more conducive to healthy sleep. A broader cultural shift that recognizes the importance of sleep for learning, emotional regulation and health is needed to help adolescents meet their biological sleep needs.

The present study has several strengths, including the use of objective measures of sleep, standardized cognitive tests, a before/after study design with a control group and a randomized allocation ensuring baseline comparability. However, the relatively small sample size, particularly for actigraphy analyses due to compliance issues, should be acknowledged. The actigraphy sample size was reduced due to compliance issues, particularly among boys and participants in the Control-SST group, possibly reflecting lower engagement in the absence of intervention benefit. This attrition can introduce a possible selection bias, although baseline comparisons did not differ significantly between included and excluded participants. Additionally, subgroup analyses (e.g., by chronotype) lacked statistical power.

Another limitation is that sleep analyses focused exclusively on school nights. Although this choice was aligned with the primary objective of the intervention, which specifically targeted weekday sleep restriction, it prevented us from examining potential changes in weekend sleep patterns. We could not assess whether compensatory weekend sleep or social jetlag (i.e., the discrepancy between weekday and weekend sleep timing) were modified by the intervention. These aspects are important, as both weekend catch-up sleep and social jetlag have been linked to physical and mental health outcomes in adolescents^24^. Future studies using more complete longitudinal sleep recordings should aim to capture these dynamics to provide a more comprehensive understanding of the effects of delayed school start times.

Another limitation is that subjective sleep parameters derived from sleep diaries were not analysed in the present study. Sleep diaries were primarily used to support actigraphy scoring by defining sleep intervals. Although objective measures provide robust estimates of sleep duration, subjective perceptions of sleep may not always align with actigraphic measurements and can offer complementary insights into adolescents’ sleep experience.

As previous studies have experienced, recruiting schools to participate in a randomized study which implies modification of schedules is particularly difficult^20^. The controlled boarding school setting of our study, while controlling for environmental variability and commute facilities, may limit generalizability to typical day school populations. This setting provided a relatively structured environment that may have reduced variability in evening routines compared with typical home settings. Dinner times were fixed and lights-out was scheduled at 21:30, with students generally expected to limit electronic device use afterward, although adherence to these rules may vary. Students also slept in shared dormitory rooms, which may have contributed to more synchronized sleep schedules. This structured context likely strengthened the internal validity of the study by reducing variability in evening activities and helping isolate the effect of the school start time intervention. However, it may limit generalizability to typical day-school environments, where parental supervision, extracurricular activities, and evening screen exposure vary more widely.

Reflecting a strong scientific consensus, major professional organizations including the National Sleep Foundation^55^, the American Academy of Sleep Medicine^56^ and the American Academy of Pediatrics^57^, recommend school start times of 8:30 a.m. or later for adolescents. Only about 18% of high schools in the USA meet this recommendation^58^. In France, middle school uniformly starts at 8 a.m. However, these recommendations remain relatively pragmatic, representing a compromise between biological considerations and logistical constraints, although some studies have suggested that even later start times (e.g., 10:00 a.m.) may provide additional benefits^59^.

The primary barriers to implementation are logistical rather than scientific, with school districts citing concerns about transportation, after-school activities and families schedules^60^. This gap between evidence and practice illustrates how operational constraints can impede the translation of sleep research into educational practice, leaving many adolescents with chronic sleep deprivation despite effective solutions. Interestingly, delayed start times have also been associated with improved sleep in parents^61^ and teachers^62^, and parent-teacher associations are increasingly advocating a delay in school^63^. Whether this growing support from advocacy groups will be enough to overcome long-standing logistical challenges and lead to widespread policy change remains an open question.

In conclusion, this controlled trial provides compelling evidence that a one-hour delay in school start time significantly improves adolescent sleep duration and daytime functioning. The intervention effectively counteracted the natural developmental decline in sleep without delaying bedtimes, resulting in both objective sleep improvements and enhanced cognitive performance, particularly for inhibitory control. These findings add to the growing scientific consensus supporting later school start times as a public health strategy to mitigate sleep deprivation in adolescents. Educational policymakers should consider these benefits when weighing the logistical challenges of implementing delayed start times against the potential improvements in student well-being and cognitive functioning.

## Supplementary Information

Supplementary Information.

## Data Availability

The datasets generated during and/or analysed during the current study are available from the corresponding author on reasonable request.
