# Supplementary material for: A one-hour delayed school start improves sleep, sleepiness and inhibitory control in early adolescents in a randomized controlled trial
Source: Sci Rep. 2026 May 15;16:22215. doi: 10.1038/s41598-026-50892-6 (PMC13369917; doi:10.1038/s41598-026-50892-6)
Supplement: Supplementary file 1 — Supplementary Information. [file 41598_2026_50892_MOESM1_ESM.docx]

|  | Actigraphy | |  | Questionnaires and cognitive tasks | |  |
| --- | --- | --- | --- | --- | --- | --- |
|  | Included (N=50) | Excluded  (N=36) | p | Included (N=73) | Excluded  (N=13) | p |
| Age (year) | 12.8 (0.7) | 12.7 (0.6) | 0.923 | 12.7 (0.6) | 12.8 (0.7) | 0.519 |
| Gender (girl) | 68.0% (34) | 36.1% (13) | 0.003 | 49.3%(36) | 69.2% (9) | 0.185 |
| Scholarship (yes) | 48.0% (24) | 50.0% (18) | 0.855 | 50.7% (37) | 38.5% (5) | 0.417 |
| Group (Delayed-SST) | 60.0% (30) | 41.7% (15) | 0.093 | 49.3% (36) | 69.2% (9) | 0.185 |

Supplementary Table. Socio-demographic differences between included and excluded participants

Age is presented as *Mean (SD)* and compared between included and excluded participants using the Mann–Whitney U test. Binary variables are reported as *percentage (N)* and compared using the chi-square test*.*
